# The RNA‐ and DNA‐Binding Protein Y‐Box Binding Protein 1 (YB‐1) Regulates *i*NKT Cell Development

**DOI:** 10.1002/eji.70201

**Published:** 2026-05-19

**Authors:** Silvia Schulze, Laura Knop, Nouria Jantz‐Naeem, Vladyslava Dovhan, Stephan Fricke, Sascha Kahlfuß, Thomas Schüler, Ursula Bommhardt

**Affiliations:** ^1^ Institute of Clinical Immunology and Cell Therapeutics Medical Faculty Otto‐von‐Guericke‐University Magdeburg Magdeburg Germany; ^2^ Health Campus Immunology, Infectiology and Inflammation (GCI^3^), Medical Faculty Otto‐von‐Guericke‐University Magdeburg Magdeburg Germany; ^3^ Fraunhofer Institute for Cell Therapy and Immunology Perlickstr Leipzig Germany; ^4^ Institute of Medical Microbiology and Hospital Hygiene, Medical Faculty Otto‐von‐Guericke‐University Magdeburg Magdeburg Germany; ^5^ Center For Health and Medical Prevention (CHaMP) Otto‐von‐Guericke‐University Magdeburg Germany

**Keywords:** apoptosis, *i*NKT cells, TCR signaling, thymus, YB‐1

## Abstract

Y‐box binding protein 1 (YB‐1) is a multifunctional RNA‐ and DNA‐binding protein with broad regulatory functions in gene expression, particularly at the posttranscriptional level. Here, we demonstrate that conditional deletion of YB‐1 at the double‐positive (DP) thymocyte stage causes an ∼80% reduction of invariant natural killer T (*i*NKT) cells in thymus, spleen, and liver, evident already in day‐14 neonates and persisting into adulthood. Our data reveal CD44^−^NK1.1^−^ stage 1 accumulation and a selective loss of CD44^+^NK1.1^+^ stage 3 *i*NKT cells, indicating a postselection maturation defect. All *i*NKT cell subsets (*i*NKT1, *i*NKT2, *i*NKT17) were reduced, with thymic *i*NKT1 and splenic *i*NKT17 cells most severely affected. PMA/ionomycin‐stimulated YB‐1‐deficient *i*NKT cells showed preserved IFN‐γ^+^/IL‐4^+^ frequencies but reduced per‐cell cytokine production and a loss of IL‐17 production. Interestingly, YB‐1^KO^ DP thymocytes showed increased CD1d levels, suggesting increased TCR signal strength in the thymus of YB‐1‐deficient mice. Whereas CD5 levels were elevated, basal Nur77, ICOS, and CD122 (IL‐15Rβ) expression were reduced in *i*NKT cells. Furthermore, apoptosis was increased, particularly at *i*NKT stages 2–3. Together, these findings identify YB‐1 as a central regulator of *i*NKT cell development that integrates TCR, co‐stimulatory, and IL‐15 signaling to ensure postselection *i*NKT cell maturation, effector subset specification, and survival.

## Graphical abstract

YB‐1 is essential for invariant NKT cell development, integrating TCR, co‐stimulatory, and IL‐15 signaling to control maturation, subset differentiation, and survival. Its loss at the double‐positive thymocyte stage causes ∼80% iNKT cell depletion, maturation arrest, impaired cytokine production, and increased apoptosis.

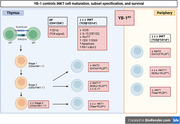

**Abbreviations**
Bcl‐2: B‐cell lymphoma 2
CD1d‐Tet: murine CD1d tetramer loaded with PBS‐57
DP: double‐positive
ICOS: inducible T‐cell costimulator
IFN‐γ: interferon‐gamma
IL: interleukin
*i*NKT cell: invariant natural killer T cell
MFI: mean fluorescence intensity
PD‐1: programmed cell death protein 1
PI: propidium iodide
PLZF: promyelocytic leukemia zinc finger
RORγt: RAR‐related orphan receptor gamma t
SLAM: signaling lymphocyte activation molecule
T‐bet: T‐box expressed in T cells
TCR: T‐cell receptor
TF: transcription factor
YB‐1: Y‐box binding protein 1

## 1 Introduction

Invariant natural killer T (*i*NKT) cells are a major subset of innate‐like T lymphocytes that bridge innate and adaptive immunity. In mice, *i*NKT cells express a semi‐invariant T cell receptor (TCR) composed of a canonical Vα14‐Jα18 α‐chain paired with a limited repertoire of β‐chains (Vβ2, Vβ7, or Vβ8). This TCR specifically recognizes glycolipid antigens presented by the nonpolymorphic major histocompatibility complex class I‐like molecule CD1d [1, 2]. Despite their restricted TCR diversity, *i*NKT cells can recognize a broad range of self and microbial lipid antigens, which are widely distributed in cellular membranes. For instance, *i*NKT cells recognize the sponge‐derived glycolipid α‐galactosylceramide or its analog PBS‐57 with high affinity, which can be used to identify *i*NKT cells [3, 4, 5]. Upon activation, *i*NKT cells rapidly secrete large amounts of cytokines, including interleukin (IL)‐4, IL‐17, and interferon‐gamma (IFN‐γ), and thus play a pivotal role in shaping early immune responses [6]. Although constituting a relatively small proportion of lymphocytes, *i*NKT cells critically contribute to host defenses against infections and are involved in the pathogenesis of autoimmune diseases and allergies [7]. *i*NKT cells also act as central regulators of antitumor immunity, where their quantitative and functional deficits in cancer patients associate with diminished antitumor immune responses. By directly killing tumor cells, remodeling the tumor microenvironment, and demonstrating superior tumor infiltration and motility, *i*NKT cells are emerging as a promising platform for next‐generation cellular immunotherapy [8, 9].

*i*NKT cell development diverges from conventional α/β T cells at the CD4^+^CD8^+^ double‐positive (DP) stage in the thymus. Rather than interacting with thymic epithelial cells, *i*NKT cells undergo positive selection via CD1d‐expressing DP thymocytes that co‐express signaling lymphocyte activation molecule (SLAM) receptors [10, 11]. Thymic maturation of *i*NKT cells was initially described as a linear progression through four stages (stage 0–3), defined by expression of surface markers such as CD44 and NK1.1 [12]. More recent models describe functional lineage diversification into three main subsets: *i*NKT1, *i*NKT2, and *i*NKT17, distinguished by the expression of the master transcription factor (TF) promyelocytic leukemia zinc finger (PLZF), lineage‐defining TFs, and their cytokine profiles [13, 14, 15]. *i*NKT1 cells (T‐bet^+^PLZF^lo^) produce IFN‐γ, *i*NKT2 cells (GATA3^+^PLZF^hi^) secrete IL‐4, and *i*NKT17 cells (RAR‐related orphan receptor gamma (RORγt)^+^PLZF^int^) primarily produce IL‐17. These subsets populate peripheral organs as pre‐armed effector cells and contribute to immune regulation in tissue‐specific contexts [16, 17]. Both intrinsic and extrinsic signals shape *i*NKT cell development and homeostasis. These encompass a tightly controlled TCR signaling strength [18, 19, 20], homotypic interactions of SLAM family receptors that provide signals for activation of the Src family kinase Fyn [11, 21], co‐stimulatory signals via CD28‐CD80/86 [22, 23], and inducible T cell costimulator (ICOS)‐ICOS‐L interactions [24]. Besides the master TF PLZF and early growth response 2 (Egr2), other TF such as c‐Myc [25, 26], Runx1, T‐bet, and GATA3, are essential players at distinct stages of lineage commitment, proliferation, and functional polarization [27]. *i*NKT cell development also depends on the cytokine IL‐15 [28, 29, 30], whereas peripheral *i*NKT cell homeostasis is driven by IL‐7 receptor signals [31]. Nevertheless, the regulators that integrate these and other signals remain incompletely defined.

Y‐box binding protein 1 (YB‐1) is a highly conserved cold‐shock domain‐containing protein exerting pleiotropic functions in RNA and DNA metabolism, including mRNA stabilization, translation, and gene expression [32, 33, 34]. As a component of messenger ribonucleoprotein particles (mRNPs), YB‐1 is predominantly localized in the cytoplasm, where it protects mRNA from exonucleolytic degradation and modulates translation in a concentration‐dependent manner. Upon cellular stress, phosphorylated YB‐1 translocates to the nucleus, where it regulates gene transcription [35, 36, 37]. YB‐1 has been implicated in tumorigenesis and serves as a therapeutic target in several malignancies [38, 39]. It is also expressed and activated in T cells, where it was associated with cell proliferation and survival [40, 41]. YB‐1's role in T cells, particularly in T cell development, however, remains poorly understood. Here, we explored the function of YB‐1 in iNKT cells using conditional Ybx1 knockout mice expressing the recombinase Cre specifically in CD4+ cells. We show that YB‐1 is indispensable for *i*NKT cell development as YB‐1 deficiency leads to a profound loss of all *i*NKT cell subsets in the thymus and peripheral organs. These findings identify YB‐1 as a novel key regulator of *i*NKT cell development.

## 2 Results

### 2.1 *i*NKT Cell Development Is Defective in Absence of YB‐1

**FIGURE 1:**
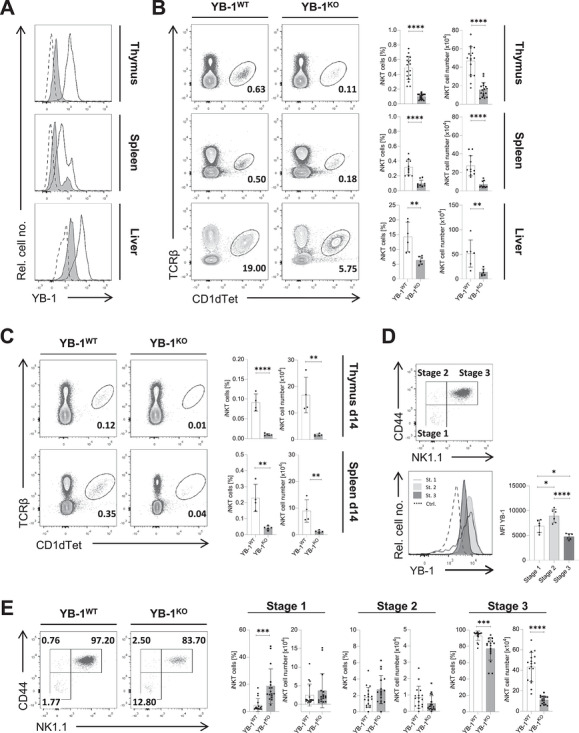
Defective *i*NKT cell development in YB‐1^KO^ mice. (A) TCRβ^+^CD1dTet^+^ *i*NKT cells from YB‐1 wild‐type (WT, black line) and YB‐1 knockout (KO, shaded histogram) mice were analyzed by flow cytometry for YB‐1 expression. A staining control using secondary antibody only is shown in a dashed line. Data are representative of two independent experiments for thymus and spleen (total *n* = 7/genotype) and one experiment for liver (*n* = 1/genotype). (B) Representative flow cytometry contour plots show the frequency of TCRβ^+^CD1dTet^+^ *i*NKT cells in the thymus, spleen, and liver in YB‐1^WT^ and YB‐1^KO^ mice. Bar graphs summarize frequencies and absolute numbers of *i*NKT cells across the indicated organs and experimental groups. Data are representative of two to five independent experiments (total *n* = 6–17/group). (C) Thymi and spleens of neonatal (day 14) YB‐1^WT^ and YB‐1^KO^ mice were analyzed by flow cytometry. Representative contour plots show the frequency of TCRβ^+^CD1dTet^+^ *i*NKT cells in both groups. Bar graphs summarize frequencies and absolute numbers of *i*NKT cells across the respective organs and experimental groups. Data are representative of two independent experiments (total *n* = 4–5/group). (D) Stage‐specific YB‐1 expression in thymic *i*NKT cells from C57BL/6 mice was analyzed by flow cytometry. Representative dot plot shows the gating strategy used to define maturation stages of live, TCRβ^+^CD1dTet^+^ *i*NKT cells based on CD44 and NK1.1 expression. The bar graph summarizes YB‐1 mean fluorescence intensity (MFI) across the indicated maturation stages (stage 1: CD44^−^NK1.1^−^; stage 2: CD44^+^NK1.1^−^; stage 3: CD44^+^NK1.1^+^) (total *n* = 6 acquired in one experiment). Statistical significance was determined using one‐way ANOVA. (E) Thymic TCRβ^+^CD1dTet^+^ *i*NKT cells from YB‐1^WT^ and YB‐1^KO^ mice were analyzed by flow cytometry for maturation stages defined by CD44 and NK1.1 expression. Percentages and cell numbers are shown in representative dot plots and corresponding bar graphs (five independent experiments with total *n* = 17/group). (B–E) Bar graphs show mean values ± SD and statistical significances (*p ≤ 0.05; **p ≤ 0.01; ***p ≤ 0.001; ****p ≤ 0.0001).

To investigate the role of YB‐1 in *i*NKT cell development, we generated conditional YB‐1 knockout mice (*Ybx1* ^flx/flx^ × *Cd4*‐Cre, hereafter referred to as YB‐1^KO^) and used *Ybx1* ^wt/wt^ × *Cd4*‐Cre mice as wild‐type (WT) controls. Efficient deletion of *Ybx1* in thymic and peripheral *i*NKT cells was confirmed by intracellular flow cytometry, which showed loss of YB‐1 protein in TCRβ^+^CD1d‐tetramer^+^ (CD1dTet^+^) *i*NKT cells from YB‐1^KO^ mice (Figure 1A). In adult mice, the frequency and absolute number of thymic *i*NKT cells were reduced (∼80%) in YB‐1^KO^ mice compared with WT controls. A similar reduction was also observed in the spleen and liver (Figure 1B). To determine whether this defect is already evident in early life, we analyzed *i*NKT cell frequencies and cell numbers in day‐14 neonatal mice. Strikingly, the loss of *i*NKT cells was even more pronounced in YB‐1^KO^ neonates compared with adults. In both the thymus and spleen of neonatal YB‐1^KO^ mice, *i*NKT cells were nearly absent (Figure 1C), indicating that YB‐1 is essential at very early stages of *i*NKT cell ontogeny, and this deficit is not compensated during postnatal development.

According to the ‘linear model’ of *i*NKT cell development [12], different *i*NKT cells are discriminated by CD44 and NK1.1 expression, subdividing them into stage 1 (CD44^−^NK1.1^−^), stage 2 (CD44^+^NK1.1^−^), and stage 3 (CD44^+^NK1.1^+^). To determine whether YB‐1 levels correlate with the YB‐1‐dependency of specific *i*NKT cell subtypes, we quantified YB‐1 levels in thymic *i*NKT cells. Interestingly, YB‐1 was differentially expressed among the different stages, with the highest expression in stage 2 *i*NKT cells (Figure 1D). This stage‐specific expression pattern suggests that YB‐1 may exert distinct functions during *i*NKT cell maturation.

Flow cytometric analysis showed a significant accumulation of stage 1 *i*NKT cells in YB‐1^KO^ mice, along with a marked reduction in stage 3 frequencies. Quantification of absolute cell numbers confirmed a selective and significant decrease in stage 3 *i*NKT cells in the absence of YB‐1, whereas earlier stages remained unaffected (Figure 1E). These data suggest that YB‐1 is dispensable for initial *i*NKT cell lineage selection but required for maturation of thymic *i*NKT cells beyond early developmental stages, in particular for CD44^+^NK1.1^+^ *i*NKT cells.

### 2.2 YB‐1 Influences the Composition of *i*NKT Cell Subsets and Cytokine Production

**FIGURE 2:**
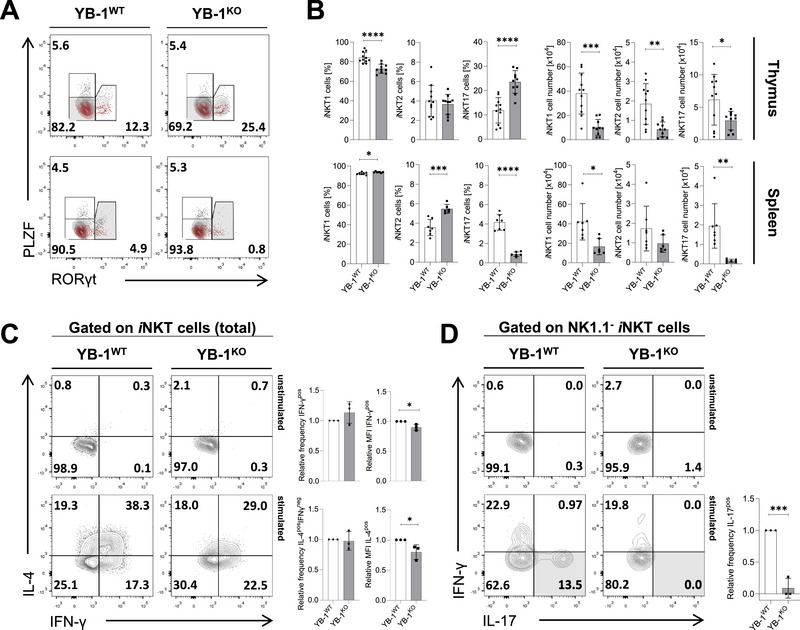
YB‐1 affects the distribution and function of *i*NKT cell subsets. (A, B) Thymic and splenic TCRβ^+^CD1dTet^+^ *i*NKT cells from YB‐1^WT^ and YB‐1^KO^ mice were analyzed by flow cytometry after intracellular staining for PLZF and RORγt to identify *i*NKT cell subsets. (A) Numbers adjacent to gated populations indicate the frequencies of PLZF^lo^RORγt^−^ (*i*NKT1; bottom left), PLZF^hi^RORγt^−^ (*i*NKT2; top left), and PLZF^int^RORγt^+^ (*i*NKT17; right) cells. The PLZF fluorescence minus one (FMO) control is shown in red. (B) Bar graphs summarize the frequencies and absolute numbers of *i*NKT cell subsets in the thymus (three independent experiments with total *n* = 10–11/group) and spleen (two independent experiments with total *n* = 6–7/group) of YB‐1^WT^ and YB‐1^KO^ mice. (C, D) Enriched TCRβ^+^CD1dTet^+^ thymocytes from YB‐1^WT^ and YB‐1^KO^ mice were stimulated with PMA/ionomycin, and gated TCRβ^+^CD1dTet^+^ *i*NKT cells were analyzed by flow cytometry for intracellular cytokine production. Representative contour plots show cytokine expression profiles for each group. Bar graphs summarize the relative frequencies and MFIs of cytokine‐producing *i*NKT cells. For each data point, two thymi were pooled per genotype. In total, three data points were acquired in three independent experiments. (B–D) Bar graphs show mean values ± SD; *p ≤ 0.05; **p ≤ 0.01; ***p ≤ 0.001; ****p ≤ 0.0001. For relative frequencies, individual values were normalized to the mean of the YB‐1^WT^ group.

To investigate the effect of YB‐1‐deficiency on effector subset differentiation, we performed intracellular staining for PLZF and RORγt in TCRβ^+^CD1dTet^+^ *i*NKT cells. Based on TF profiles, *i*NKT cells were categorized into *i*NKT1 (PLZF^lo^RORγt^−^), *i*NKT2 (PLZF^hi^RORγt^−^), and *i*NKT17 (PLZF^int^RORγt^+^) cells. Subset analysis revealed a pronounced decrease in the frequency of thymic *i*NKT1 cells in YB‐1^KO^ mice, accompanied by an increased frequency of *i*NKT17 cells (Figure 2A,B). This shift in subset composition aligns with the observed reduction of thymic CD44^+^NK1.1^+^ *i*NKT cells (Figure 1E), as *i*NKT1 cells are the only subset expressing NK1.1 under steady‐state conditions [16]. In the spleen of YB‐1^KO^ mice, the frequency of *i*NKT17 cells was markedly reduced (∼75%), and the frequencies of *i*NKT1 and *i*NKT2 cell subsets were increased (Figure 2A,B). However, analysis of absolute cell numbers revealed that almost all *i*NKT cell subsets were strongly reduced in both thymus and spleen (Figure 2A,B). This suggests that YB‐1 is required for the generation of all effector *i*NKT cell lineages, with thymic *i*NKT1 cells and splenic *i*NKT17 cells being most affected by the loss of YB‐1.

*i*NKT cells are characterized by rapid cytokine secretion upon activation. Each subset exhibits a distinct cytokine profile: *i*NKT1 cells (corresponding to CD44^+^NK1.1^+^ stage 3 cells) primarily produce IFN‐γ and low levels of IL‐4, *i*NKT2 cells mainly produce IL‐4, and *i*NKT17 cells secrete IL‐17 upon stimulation [7]. To assess whether YB‐1 influences this functional polarization, we stimulated thymic *i*NKT cells with PMA and ionomycin, followed by intracellular cytokine staining and flow cytometric analysis. Upon stimulation, the frequencies of IFN‐γ^+^ *i*NKT1 and IL‐4+IFN‐γ‐ *i*NKT2 cells were comparable in YB‐1^WT^ and YB‐1^KO^ *i*NKT cells. However, the production of both cytokines per cell was reduced in YB‐1^KO^ *i*NKT cells (Figure 2C). Furthermore, in YB‐1^WT^ thymocytes, a clear population of IL‐17‐producing *i*NKT cells was detectable, whereas IL‐17^+^ *i*NKT cells were nearly absent in YB‐1‐deficient thymocytes (Figure 2D). These results show that loss of YB‐1 is associated with impaired cytokine production, with particularly strong effects on IL‐17‐producing *i*NKT17 cells.

### 2.3 YB‐1 Modulates TCR Signaling and Defines the Phenotype of *i*NKT Cells

**FIGURE 3:**
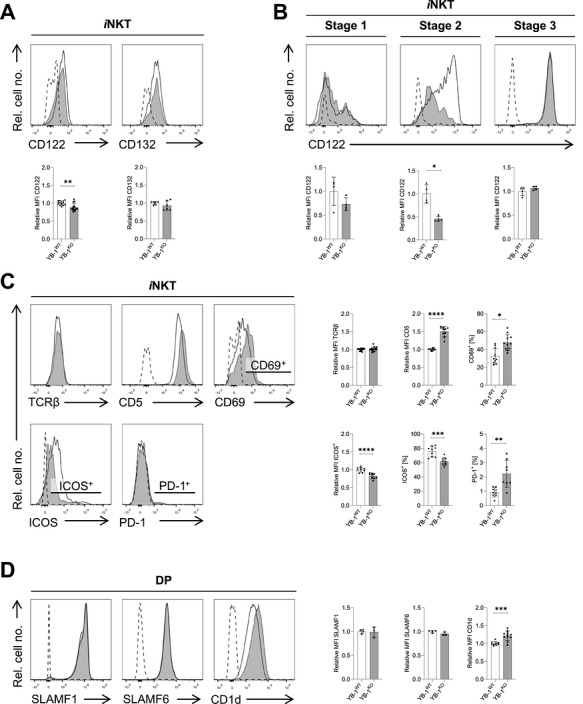
YB‐1‐deficiency alters the phenotype of *i*NKT cells and CD1d expression on DP thymocytes. (A, C) Thymic TCRβ^+^CD1dTet^+^ *i*NKT cells were analyzed by flow cytometry for expression of the indicated surface markers. Bar graphs summarize relative MFIs or frequencies across experimental groups (total *n* = 6–17/group acquired in two to five independent experiments). Relative MFI for CD132 was determined on CD44^hi^ *i*NKT cells. (B) Stage‐specific CD122 expression in thymic TCRβ^+^CD1dTet^+^ *i*NKT cells was analyzed by flow cytometry. Maturation stages 1–3 were defined based on CD44 and NK1.1 expression, as in Figure 1D. Bar graphs summarize relative MFIs (total *n* = 4/group acquired in one experiment). (D) Live, CD4^+^CD8^+^ DP thymocytes were analyzed for expression of SLAMF1, SLAMF6 (*n* = 3–4/group acquired in one experiment), and CD1d (*n* = 10–11/group acquired in three independent experiments) by flow cytometry. Bar graphs summarize relative MFIs. (A–D) Representative histograms show marker expression in YB‐1^WT^ (black line) and YB‐1^KO^ (shaded histogram) mice. FMO or isotype controls are shown as dashed lines. Bar graphs show mean values ± SD; *p ≤ 0.05; **p ≤ 0.01; ***p ≤ 0.001; ****p ≤ 0.0001. For relative MFIs, individual values were normalized to the mean of the YB‐1^WT^ group. Statistical significance for panels (B–D) was determined using the Mann–Whitney *U* test.

IL‐15 produced by medullary thymic epithelial cells is essential for *i*NKT cell survival and maturation [29, 30]. Accordingly, we further examined IL‐15 receptor expression. In YB‐1^KO^ mice, the expression of CD122 (IL‐15Rβ chain) was significantly reduced on *i*NKT cells, whereas CD132 (common γ‐chain) levels remained unchanged (Figure 3A). A more detailed analysis of CD122 expression revealed that CD122 levels on stage 1 and 3 *i*NKT cells were comparable between both genotypes, while there was a strong reduction of CD122 on stage 2 *i*NKT cells in the absence of YB‐1 (Figure 3B). This suggests a stage‐specific contribution of YB‐1 to IL‐15‐dependent signaling in *i*NKT cells.

Besides cytokine receptor signals, TCR triggering is crucial for *i*NKT cell development [18, 42]. To test whether the abundance of surface TCR molecules is affected by YB‐1, TCRβ expression was analyzed. TCRβ expression levels did not differ significantly between YB‐1^WT^ and YB‐1^KO^ *i*NKT cells (Figure 3C). In contrast, CD5 levels and the frequency of CD69^+^ *i*NKT cells, reflecting cumulative TCR signal strength during thymic development [43] and recent TCR activation events associated with thymocyte selection [44], respectively, were significantly elevated in YB‐1^KO^ *i*NKT cells (Figure 3C).

**FIGURE 4:**
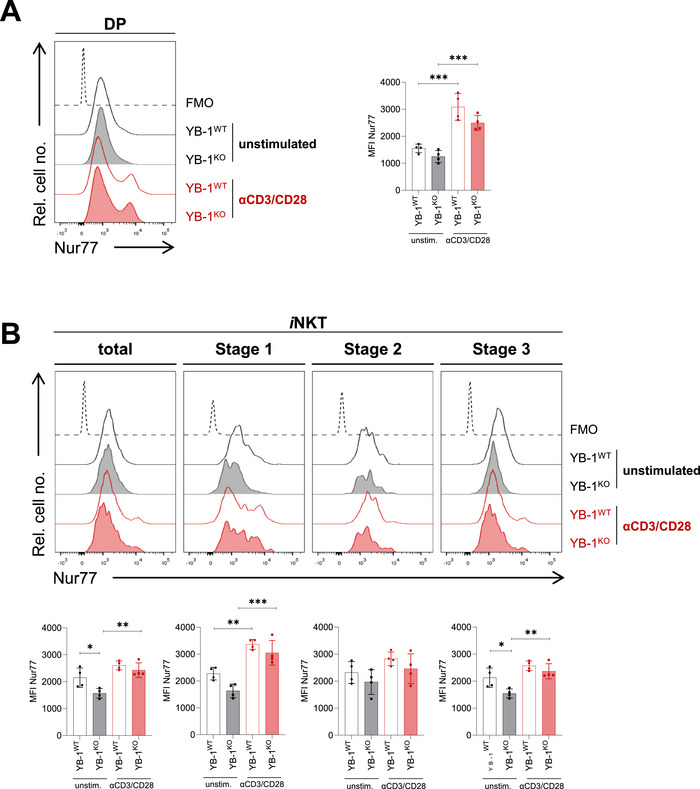
Nur77 expression is reduced in YB‐1‐deficient *i*NKT cells in the steady‐state. (A) Live, CD4^+^CD8^+^ DP thymocytes from YB‐1^WT^ and YB‐1^KO^ mice were analyzed by flow cytometry for expression of Nur77 under steady‐state conditions or after 2 h stimulation with CD3/CD28 antibodies. A representative overlaid histogram shows Nur77 expression in unstimulated and CD3/CD28‐stimulated DP thymocytes from YB‐1^WT^ and YB‐1^KO^ mice, including FMO control. The bar graph summarizes MFIs across experimental groups. (B) Stage‐specific Nur77 expression in live thymic TCRβ^+^CD1dTet^+^ *i*NKT cells from YB‐1^WT^ and YB‐1^KO^ mice was analyzed by flow cytometry under steady‐state conditions or after CD3/CD28 stimulation. Maturation stages 1–3 were defined based on CD44 and NK1.1 expression, as in Figure 1D. Representative overlaid histograms and bar graphs are shown as in (A), summarizing MFIs across experimental groups. (A, B) Bar graphs show mean values ± SD; *p ≤ 0.05; **p ≤ 0.01; ***p ≤ 0.001. Statistical significance was determined using two‐way ANOVA with Tukey's multiple comparison test (*n* = 4/group acquired in one experiment).

We further examined the expression of ICOS and programmed cell death protein 1 (PD‐1), two CD28‐family co‐receptors expressed on *i*NKT cells. While ICOS provides stimulatory signals, PD‐1 is inhibitory and promotes apoptosis [27]. YB‐1^KO^ *i*NKT cells showed a markedly reduced ICOS expression and an increased proportion of PD‐1^+^ cells (Figure 3C), indicating a shift toward a more pro‐apoptotic phenotype. Moreover, *i*NKT cell development requires SLAM receptor signaling, with SLAMF1 and SLAMF6 being the most relevant receptors [11]. SLAMF1 and SLAMF6 expression on DP cells was comparable between genotypes (Figure 3D). However, CD1d levels were clearly increased on YB‐1‐deficient DP thymocytes (Figure 3D), suggesting increased TCR signal strength in the thymus of YB‐1‐deficient mice. To validate this assumption, we next analyzed Nur77 expression as a reporter of TCR signaling strength [18, 42, 45]. In unstimulated and CD3/CD28‐activated DP thymocytes, Nur77 expression was similar between YB‐1^WT^ and YB‐1^KO^ mice, with only a slight, nonsignificant reduction in YB‐1^KO^ DP thymocytes (Figure 4A). In contrast, unstimulated total and stage 3 *i*NKT cells from YB‐1^KO^ mice showed a significant reduction in Nur77 expression compared with YB‐1^WT^, whereas Nur77 levels after CD3/CD28 stimulation were comparable between genotypes (Figure 4B). Taken together, these results indicate that remaining *i*NKT cells from YB‐1^KO^ mice exhibit reduced tonic TCR signaling under steady‐state conditions, particularly within the stage 3 *i*NKT cell population.

### 2.4 YB‐1 Promotes *i*NKT Cell Survival

**FIGURE 5:**
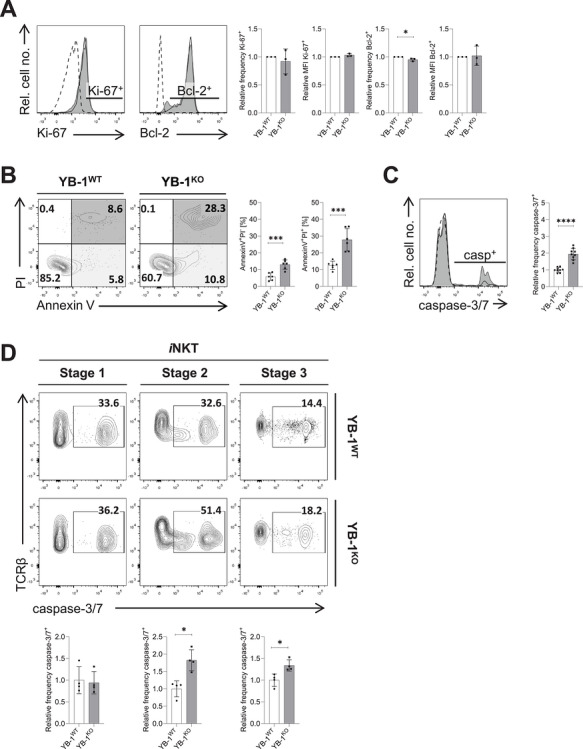
Increased apoptosis in YB‐1‐deficient *i*NKT cells. (A) Thymic TCRβ^+^CD1dTet^+^ *i*NKT cells from YB‐1^WT^ (black line) and YB‐1^KO^ (shaded histogram) mice were analyzed by flow cytometry for intracellular expression of Ki‐67 and Bcl‐2, shown as representative histograms. FMO or isotype controls are indicated by dashed lines. The gating strategy used to define positive cell populations is indicated. Corresponding bar graphs show relative MFIs or frequencies across experimental groups. For each data point, 2–3 thymi were pooled per genotype. In total, three data points were acquired in three independent experiments. (B) Apoptosis of thymic TCRβ^+^CD1dTet^+^ *i*NKT cells was assessed by Annexin V/ propidium iodide (PI) staining. Representative dot plots show early (Annexin V^+^PI^−^) and late (Annexin V^+^PI^+^) apoptotic *i*NKT cells from YB‐1^WT^ and YB‐1^KO^ mice. Bar graphs summarize results from two independent experiments, total *n* = 6/group. (C) Apoptosis of thymic TCRβ^+^CD1dTet^+^ *i*NKT cells was analyzed by staining for cleaved active caspase‐3/7. Colors as described in (A). Data are representative of three independent experiments, total *n* = 9/group. (D) Cleaved caspase‐3/7 expression was analyzed in thymic TCRβ^+^CD1dTet^+^ *i*NKT cells across maturation stages defined by CD44 and NK1.1 expression, as in Figure 1D. Representative contour plots show expression of activated caspase‐3/7 in *i*NKT cells from YB‐1^WT^ and YB‐1^KO^ mice, with percentages of caspase‐3/7^+^ cells indicated. Bar graphs summarize the relative frequencies of caspase‐3/7^+^ *i*NKT cells (total *n* = 4/group acquired in one experiment). Statistical significance was determined using the Mann–Whitney *U* test. (A–D) Bar graphs show mean values ± SD; *p ≤ 0.05; ***p ≤ 0.001; ****p ≤ 0.0001. For relative frequencies or MFIs, individual values were normalized to the mean of the YB‐1^WT^ group.

To address whether reduced *i*NKT cell numbers in YB‐1^KO^ mice reflect defects in proliferation or survival, we analyzed *i*NKT cell division and apoptosis by flow cytometry. The proportion of Ki‐67^+^ *i*NKT cells did not differ between YB‐1^WT^ and YB‐1^KO^ mice (Figure 5A), suggesting that proliferative capacity is preserved. In contrast, the frequency of Bcl‐2^high^ *i*NKT cells was lower in YB‐1^KO^ mice (Figure 5A). To directly evaluate apoptosis, we performed staining with Annexin V/propidium iodide (PI) and a probe recognizing activated caspases 3 and 7. Both early (Annexin V^+^PI^−^) and late (Annexin V^+^PI^+^) apoptotic *i*NKT cells were significantly increased in YB‐1^KO^ mice (Figure 5B). Consistently, the percentage of activated caspase‐3/7^+^ *i*NKT cells was elevated in YB‐1^KO^ compared with YB‐1^WT^ mice (Figure 5C). Stage‐resolved analysis showed increased levels of activated caspase‐3/7 in stage 2 and stage 3 *i*NKT cells (Figure 5D). These findings indicate that the reduced *i*NKT cell pool in YB‐1‐deficient mice is primarily due to impaired survival rather than defective proliferation.

## 3 Discussion

Thymic development of *i*NKT cells requires precise coordination of TCR signaling, transcriptional programming, and cytokine‐mediated survival [17, 29, 42]. In the present study, we identify the RNA‐ and DNA‐binding protein YB‐1 as an essential factor for *i*NKT cell ontogeny, as its deletion at the DP stage causes a profound and persistent loss of all major *i*NKT cell subsets in the thymus and peripheral tissues. This loss is evident as early as postnatal day 14 and persists into adulthood, indicating that YB‐1 is required for both the establishment and long‐term maintenance of *i*NKT cell populations. The analysis of PLZF and RORγt revealed that YB‐1‐deficiency leads to a global reduction in almost all thymic and splenic *i*NKT cell lineages at the level of absolute numbers, with thymic *i*NKT1 and splenic *i*NKT17 cells being most affected. This was paralleled by impaired effector function, as thymic YB‐1‐deficient *i*NKT cells displayed reduced per‐cell production of IFN‐γ and IL‐4 and a near‐complete loss of IL‐17‐producing *i*NKT17 cells, despite largely preserved frequencies of IFN‐γ^+^ and IL‐4^+^ cells. The generation of conventional Th17 cells is promoted by ICOS [46]. In our study, ICOS was also reduced in YB‐1‐deficient *i*NKT cells. Thus, the pronounced loss of IL‐17‐producing *i*NKT cells in YB‐1‐deficient mice may be linked to the reduced ICOS expression. This subset imbalance suggests that YB‐1 stabilizes lineage identity by orchestrating subset‐defining signaling networks. In addition, *i*NKT17 cell accumulation in YB‐1^KO^ thymus versus peripheral loss suggests that YB‐1 has a distinct role in either thymic maturation or peripheral homeostasis of *i*NKT17 cells. *i*NKT17 cells depend exclusively on IL‐7 for peripheral homeostasis and survival [47] and YB‐1 enhances IL‐7 responsiveness in BCP‐ALL cells by upregulating CD127 and anti‐apoptotic genes [48]. Thus, YB‐1 likely sustains *i*NKT17 cell peripheral maintenance through IL‐7 receptor signaling, with lineage tracing required to distinguish thymic versus peripheral defects. Similar subset skewing of *i*NKT cells has been reported following disruption of posttranscriptional regulators such as the RNA‐binding proteins Lin28b [49] and Roquin‐1/2 [50], as well as of mTOR‐dependent pathways [51, 52, 53, 54]. Given YB‐1's established role in regulating mRNA stability and translation [55], it is plausible that YB‐1 also coordinates both metabolic and transcriptional programs required for *i*NKT cell subset specification and function.

TCR signaling strength coordinated by SLAM receptor signaling critically influences *i*NKT cell subset differentiation [11, 19]. Positive selection *of i*NKT cells depends on strong TCR signals, reflected by high CD5 expression, which is particularly important for the differentiation of *i*NKT2 and *i*NKT17 cell subsets [18, 42, 43]. *i*NKT cell lineage commitment occurs at the DP thymocyte stage through homotypic interactions with CD1d‐ and SLAM‐expressing DP thymocytes [3, 11, 21]. SLAMF1/6 expression on DP thymocytes was unaltered in YB‐1‐deficient mice, indicating that YB‐1 does not regulate SLAM receptor expression. However, YB‐1‐deficiency was associated with increased CD1d on DP thymocytes and elevated CD5 expression on *i*NKT cells. This observation is highly relevant, as CD1d expression on DP thymocytes is a well‐established quantitative determinant of *i*NKT cell agonist selection [3, 56, 57, 58]. Previous studies have shown that increased CD1d expression on selecting thymocytes enhances the presentation of self‐lipid antigens, resulting in stronger TCR signaling during *i*NKT cell selection. Under such conditions, high‐affinity *i*NKT cell precursors undergo exaggerated negative selection, leading to reduced thymic and peripheral *i*NKT cell numbers and increased apoptosis among developing *i*NKT cells [42, 59, 60]. Accordingly, the elevated CD1d levels that we observed on DP thymocytes from YB‐1‐deficient mice provide a mechanistic explanation for several phenotypic features of the YB‐1‐deficient *i*NKT cell compartment. This includes increased CD5 expression and heightened cell death, both of which are widely accepted indicators of excessive TCR signal strength during thymic selection [19, 43]. In this context, increased CD1d expression on DP thymocytes in YB‐1‐deficient mice is also consistent with the impairment of *i*NKT1 and *i*NKT17 cell subsets observed in the thymus and periphery, arguing for altered signal calibration rather than a complete developmental arrest. Thus, we propose a model in which the heightened CD1d‐dependent signal synergizes with cell‐intrinsic requirements for YB‐1 in developing *i*NKT cells, contributing to altered subset differentiation and impaired functional maturation. As a direct read‐out for TCR signal strength, we measured Nur77 expression. Our data suggest that YB‐1 is not required for maximal, short‐term TCR signaling but rather for sustaining Nur77 expression under physiological conditions in vivo. Thus, YB‐1‐dependent TCR signaling may affect *i*NKT cell development at several levels, contributing to cell‐extrinsic and ‐intrinsic effects required for *i*NKT cell development.

YB‐1‐deficient *i*NKT cells showed no defect in proliferative capacity as measured by Ki‐67, but displayed reduced frequencies of Bcl‐2^high^ cells and increased Annexin V and activated caspase‐3/7 staining, most prominently at stage 2 and, to a lesser extent, at stage 3. These findings indicate that the reduction of the *i*NKT cell pool in the absence of YB‐1 is primarily due to impaired survival rather than defective proliferation. The conclusion that YB‐1 promotes anti‐apoptotic signaling in *i*NKT cells is consistent with observations in YB‐1‐silenced human CD4^+^ T cells [41].

Furthermore, we found that YB‐1‐deficient *i*NKT cells exhibit reduced surface expression of CD122 (IL‐15Rβ), particularly at stage 2, while expression of the common γ‐chain (CD132) was preserved. As IL‐15 trans‐presentation by medullary thymic epithelial cells is essential for the survival and maturation of stage 2 and stage 3 *i*NKT cells [29, 30, 61, 62], impaired IL‐15Rβ expression likely contributes to the block in terminal maturation and to the selective vulnerability of stage 2–3 *i*NKT cells to apoptosis. In this context, YB‐1 expression was highest in *i*NKT stage 2 cells, suggesting that YB‐1 may particularly influence signaling events relevant for stage 2 *i*NKT cell maturation.

To explore potential candidate genes linking YB‐1 to *i*NKT cell biology, we performed a literature‐based comparison of previously described YB‐1‐associated transcripts with genes implicated in *i*NKT cell development. Dong et al. identified YB‐1‐associated transcripts in MCF‐7 cells by RNA immunoprecipitation followed by transcriptomic analysis [63]. We therefore examined whether these transcripts overlap with genes reported to be involved in *i*NKT cell biology [27, 64]. The overlap between these datasets is summarized in **Table** **S1**. Importantly, although this analysis does not imply direct regulation of these genes by YB‐1 in *i*NKT cells, it highlights potential intersections that may guide future mechanistic studies.

Collectively, our results identify YB‐1 as a key factor in *i*NKT cell development. YB‐1 enables postselection *i*NKT cell precursors to survive and mature into functional *i*NKT1, *i*NKT2, and *i*NKT17 cell subsets by coordinating TCR signal integration, co‐stimulation, and cytokine receptor availability.

**Limitations and perspectives**: Although transcriptomic approaches are powerful tools to dissect T cell development, transcriptomic analyses would primarily capture the residual *i*NKT cell population that has escaped deletion or developed through alternative trajectories [65] and would not allow conclusions on lost cells. Future studies employing RNA‐binding protein immunoprecipitation and phospho‐flow analyses to specify the TCR signaling defect, and functional rescue experiments, will be required to define YB‐1's direct molecular targets and fully dissect its cell‐intrinsic roles in *i*NKT cell development.

## 4 Material and Methods

### 4.1 Mice

*Ybx1* ^flx/flx^ mice, previously described [66] and kindly provided by Dr. P. Mertens (Clinic of Nephrology and Hypertension, Diabetes and Endocrinology, Otto‐von‐Guericke‐University Magdeburg, Germany), were crossed with CD4‐Cre transgenic mice to create conditional *Ybx1* knockout (KO) mice (*Ybx1* ^flx/flx^ x CD4‐Cre). *Ybx1* ^wt/wt^ x CD4‐Cre mice were used as wild‐type (WT) controls. Mice of both sexes, aged 7–19 weeks, on a C57BL/6 (B6) background were analyzed. For assessment of early *i*NKT cell development, neonatal mice were examined at postnatal day 14. All animals were maintained in accordance with the guidelines of the Federation of European Laboratory Animal Science Associations (FELASA). Mice were housed and bred under specific pathogen‐free conditions in the Central Animal Facility of the Medical Faculty, Otto‐von‐Guericke‐University Magdeburg.

### 4.2 Antibodies

The following antibodies and reagents were used: BioLegend: Bcl‐2 (BCL/10C4), CD127 (IL‐7Rα; A7R34), CD4 (GK1.5), CD8α (53‐6.7), PD‐1 (CD279; RMP1‐14), CD44 (IM7), CD69 (H1.2F3), IFN‐γ (XMG1.2), Ki‐67 (11F6), NK1.1 (PK136), TCRβ (H57‐597), CD122 (TM‐β1), CD150/SLAMF1 (TC15‐12F12.2), SLAMF6/Ly108 (330‐AJ), isotype control (rat IgG2a, κ; RTK2758); eBioscience: CD122 (TM‐β1), ICOS (CD278; 7E.17G9), CD69 (H1.2F3), IFN‐γ (XMG1.2), IL‐17A (eBio17B7), IL‐4 (11B11), RORγt (AFKJS‐9), TCRβ (H57‐597), Nur77 (12.14); BD Pharmingen: CD4 (GK1.5), CD8a (53‐6.7), CD44 (IM7), CD5 (53‐7.3), common γ‐chain (CD132; 4G3), TCRβ (H57‐597); Invitrogen: PLZF (Mags.21F7), rabbit IgG (H+L; polyclonal). Primary antibodies against YB‐1 (D2A11; Cell Signaling Technology) were detected with PE‐conjugated F(ab')_2_ anti‐rabbit IgG (polyclonal; eBioscience) or AF647‐conjugated anti‐rabbit IgG (polyclonal donkey; BioLegend). BV421‐conjugated mouse CD1d tetramers loaded with PBS‐57 (CD1dTet) for *i*NKT cell staining were kindly provided by the Tetramer Core Facility of the National Institutes of Health.

### 4.3 Cell Preparation and Flow Cytometry

Thymocytes and splenocytes were isolated by standard procedures [67]. Red blood cells were lysed with Gey's solution when necessary. For isolation of liver lymphocytes, the liver was perfused with PBS, cut into small pieces, and gently pressed through a 200 µm metal mesh. The resulting cell suspension was washed in RPMI 1640 by centrifugation at 711 g for 5 min at 4°C, resuspended in 35% Percoll, and centrifuged again at 711 g for 7 min at 4°C without a brake. The lymphocyte pellet was collected for flow cytometric analysis. For intracellular staining of *i*NKT cell subsets, cytokines, or proliferation markers, cells were surface‐stained with anti‐TCRβ and CD1dTet [68], followed by fixation and permeabilization using the FOXP3/Transcription Factor Staining Buffer Set (eBioscience or BioLegend) according to the manufacturer's instructions. Samples were acquired on an LSRFortessa flow cytometer (BD Biosciences) and analyzed with FlowJo software version 10 (FlowJo, LLC).

### 4.4 *In Vitro* Stimulation of Thymocytes

For cytokine detection, thymic *i*NKT cells were enriched by incubating thymocytes with biotinylated CD24 antibodies and streptavidin‐conjugated magnetic beads (M1/69; BD Pharmingen). Negative selection by magnetic‐activated cell sorting (MACS) was performed with an autoMACS cell separator according to the manufacturer's instructions (Miltenyi Biotec). Enriched cells were cultured in RPMI 1640 medium supplemented with 10% FCS and stimulated with phorbol 12‐myristate 13‐acetate (PMA, 100 ng/mL) and ionomycin (1000 ng/mL) (both from Sigma‐Aldrich) for 4 h in the presence of monensin (10 µg/mL, Sigma‐Aldrich). After stimulation, cells were surface labeled with anti‐TCRβ and CD1dTet followed by fixation and intracellular staining for the indicated cytokines. For CD3/CD28 stimulation, U‐bottom 96‐well plates were coated with 50 µL of 2 µg/mL CD3 (145‐2C11, BioLegend) and 5 µg/mL CD28 antibodies (37.51, BioLegend) in PBS. Plates were incubated for 2 h at 37°C and 5% CO_2_. After washing twice with PBS, 1 × 10^6^ thymocytes were seeded in 100 µL RPMI 1640 supplemented with 10% FCS (Superior, Sigma), 1% penicillin/streptomycin (Gibco), 2 mM L‐glutamine (Gibco), 1 mM sodium pyruvate (Gibco), 0.1 mM HEPES (Gibco), 50 µM 2‐mercaptoethanol (Sigma), 1% nonessential amino acids (Gibco). Plates were rapidly centrifuged at 200 g for 1 min and incubated for 2 h at 37°C and 5% CO_2_. Viability staining was performed with Fixable Viability Dye eF780 (eBioscience) in PBS for 20 min at 4°C. Afterwards, Fc receptors were blocked using anti‐mouse CD16/32 (2.4G2 ATCC HB‐197) for 10 min at 4°C, then surface antibodies were applied for 30 min at 4°C. Intranuclear staining was conducted by fixing and permeabilizing the cells with the Foxp3/Transcription Factor Staining Buffer Set (eBioscience) overnight at 4°C, followed by a 60 min incubation with Nur77 antibody at 4°C.

### 4.5 Measurement of Apoptosis

Apoptosis was assessed by Annexin V (BioLegend) and propidium iodide (PI, Sigma‐Aldrich) staining in Annexin V binding buffer according to the manufacturer's instructions. Cleaved caspase‐3/7 was detected using the Caspase‐3/7 Green Detection Reagent (Thermo Fisher Scientific). After surface staining, cells were incubated with the reagent (1:5000 dilution) for 5 min and analyzed by flow cytometry.

### 4.6 Gene List Overlap Analysis

Gene list overlap analysis was performed using Microsoft Excel (Microsoft Corporation). A published dataset of YB‐1‐associated transcripts identified by RNA immunoprecipitation in MCF‐7 cells [63] was used as a reference and compared with gene sets implicated in *i*NKT cell development or function derived from previously published datasets [27, 64]. Genes were matched based on string correspondence, and overlapping genes were defined as those present in both datasets.

## 5 Statistical Analysis

Statistical analyses and graphical representations were performed using GraphPad Prism version 10 (GraphPad Software). Data are presented as mean ± standard deviation (SD). To calculate relative values, individual measurements from both experimental groups were normalized to the mean value of the corresponding YB‐1^WT^ group. Relative mean fluorescence intensities (MFIs) were calculated accordingly. If not indicated otherwise, statistical significance was determined using Student's unpaired two‐tailed *t*‐test. Significance levels are indicated as *p ≤ 0.05; **p ≤ 0.01; ***p ≤ 0.001; ****p ≤ 0.0001.

## Author Contributions

S.S., L.K., and U.B. designed and performed experiments. N.J.‐N. and V.D. assisted in and performed experiments. S.S., U.B., L.K., S.F., S.K., and T.S. drafted and revised the manuscript. All authors contributed to the article and approved the submitted version.

## Ethics Statement

Experimental procedures were approved by the relevant animal experimentation committee and performed in compliance with international and local animal welfare legislations (Landesverwaltungsamt Sachsen‐Anhalt Permit Number: 42502‐2‐860 UniMD, 42502‐2‐1633 UniMD).

## Conflicts of Interest

The authors declare no conflicts of interest.

## Supporting information




**Supporting File**: eji70201‐sup‐0001‐tableS1.docx.

## Data Availability

The data that support the findings of this study are available from the corresponding author upon reasonable request.
