# Supplementary material for: The RNA‐ and DNA‐Binding Protein Y‐Box Binding Protein 1 (YB‐1) Regulates iNKT Cell Development
Source: Eur J Immunol. 2026 May 19;56:e70201. doi: 10.1002/eji.70201 (PMC13185678; doi:10.1002/eji.70201)
Supplement: Supplementary file 1 — Supporting File: eji70201‐sup‐0001‐tableS1.docx. [file EJI-56-e70201-s001.docx]

**Supporting information**

**Supplementary Table 1. YB-1-associated transcripts and potential links to *i*NKT cell biology.** The table lists the top 100 YB-1-enriched transcripts identified by RNA immunoprecipitation in MCF-7 cells [1]. Genes implicated in *i*NKT cell development or function are highlighted in grey based on published datasets [2, 3]. This comparison does not imply direct regulation of these genes by YB-1 in *i*NKT cells.

| ACTN1 | ABR | FLOT1 | MRPS2 | SF3B2 |
| --- | --- | --- | --- | --- |
| ASS1 | ACTN4 | G6PD | MTA1 | SMARCA4 |
| BCL3 | AKT1 | GAA | NCOR2 | SMARCD2 |
| CCDC90A | BAT2D1 | GAK | NELF | SRRM2 |
| CTSD | BAT3 | GATAD2A | NUP62 | TAPBP |
| FAM38A | BMP7 | GLTSCR2 | PACS2 | TBX2 |
| FLNA | C12orf10 | GNA11 | PEA15 | TMEM134 |
| FOXM1 | CHMP1A | GNB2 | POLR2I | TMEM147 |
| H2AFX | CLDN3 | GRHPR | PRPF31 | TP53 |
| ITPK1 | CYB5R3 | GUK1 | PRPF6 | TSTA3 |
| JMJD3 | DPYSL2 | GYS1 | PRSS8 | TUBGCP2 |
| NME4 | EEF1A2 | HCFC1 | PXDN | UBAP2 |
| PLXND1 | EEF1D | HDLBP | RAD23A | UBC |
| PTPN6 | EEF2 | HNRPUL1 | RALY | UQCRC1 |
| SGSH | EMD | INTS1 | ROGDI | WIPF2 |
| TKT | EVL | KIAA0494 | RPLP2 | WIPI2 |
| ZYX | EXOC7 | KLHDC4 | RXRA | YBX1 |
|  | FAM134C | LPHN1 | SART1 | YLPM1 |
|  | FAM50A | MAP3K11 | SCNN1A | ZMIZ2 |
|  | FASN | MAP4 | SEMA3F | ZNF512B |
|  | FLJ14154 | MRPL12 | SEPT9 |  |

References

1 **Dong, J., Akcakanat, A., Stivers, D. N., Zhang, J., Kim, D. and Meric-Bernstam, F.**, RNA-binding specificity of Y-box protein 1. *RNA biology*. 2009. **6**: 59–64.

2 **Engel, I., Seumois, G., Chavez, L., Samaniego-Castruita, D., White, B., Chawla, A. and Mock, D. *et al.***, Innate-like functions of natural killer T cell subsets result from highly divergent gene programs. *Nature immunology*. 2016. **17**: 728–739.

3 **Shissler, S. C. and Webb, T. J.**, The ins and outs of type I iNKT cell development. *Molecular immunology*. 2019. **105**: 116–130.
